# Perianal abscess following doppler-guided transanal hemorrhoidal dearterialization

**DOI:** 10.11604/pamj.2017.28.175.13756

**Published:** 2017-10-25

**Authors:** Ahmed Bensaad, Christophe Fircket

**Affiliations:** 1 CHU Ibn Rochd, Casablanca, Morocco; 2 Hôpitaux Iris Sud Joseph Bracops, Bruxelles, Belgique

**Keywords:** Doppler-guided, transanal, hemorrhoidal dearterialization, hemorrhoids, perianal abscess

## Image in medicine

The doppler-guided transanal hemorrhoidal dearterialization (DG-THD) is a well-established and validated minimally invasive procedure for the treatment of symptomatic hemorrhoids. As compared to the excisional technique, DG-THD has the advantage of being associated with less post-operative pain and do not compromise the anatomy or the physiology of the anal canal. Our objectif is to describe the first case of perianal abscess following DG-THD. We report perianal abscess in a previously healthy 32-year-old male patient, who underwent DG-THD for grade II hemorrhoids, as an outpatient procedure. One week later, the patient complained of persistent pain in the right iliac fossa and hypogastrium. CT-scan showed a peri-rectal fluid collection with gas bubbles and peripheral enhancement. MRI was obtained after first attempt of elective drainage, showing increase of the collection diameter without any fistula, which prompted a surgical exploration and drainage. Multi sensitive E. coli was found on culture. Post-operative recovery was slow but favourable. To the best of our knowledge, no previous report of perianal abscess following DG-THD has been described in the literature, which we believe in our case to be of haematogenous origin.

**Figure 1:**
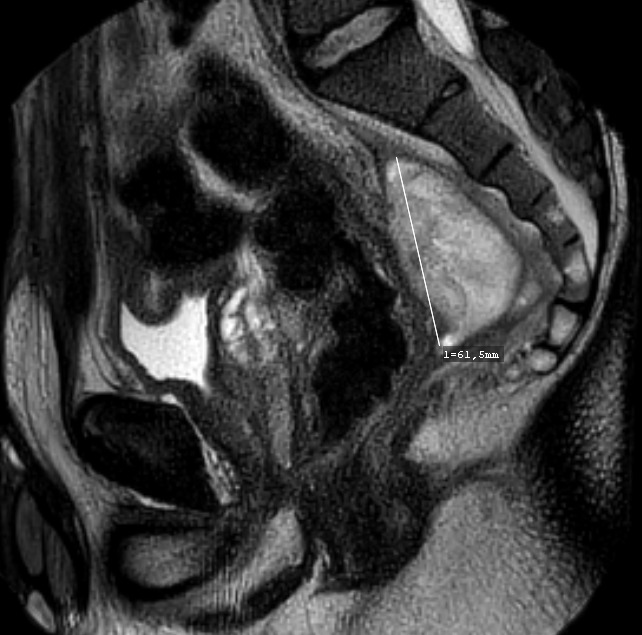
Sagittal T2-weighted pelvic magnetic resonance imaging shows perianal abscess

